# Female mice are more prone to develop an addictive-like phenotype for sugar consumption

**DOI:** 10.1038/s41598-021-86797-9

**Published:** 2021-04-01

**Authors:** Shoupeng Wei, Sarah Hertle, Rainer Spanagel, Ainhoa Bilbao

**Affiliations:** 1 Behavioral Genetics Research Group, Institute of Psychopharmacology, Central Institute of Mental Health, Heidelberg University, Mannheim, Germany; 2 Institute of Psychopharmacology, Central Institute of Mental Health, Heidelberg University, Mannheim, Germany

**Keywords:** Neuroscience, Psychology, Diseases

## Abstract

The concept of “sugar addiction” is gaining increasing attention in both the lay media and scientific literature. However, the concept of sugar addiction is controversial and only a few studies to date have attempted to determine the “addictive” properties of sugar using rigorous scientific criteria. Here we set out to systematically test the addictive properties of sugar in male and female mice using established paradigms and models from the drug addiction field. Male and female C57BL/6N (8–10 weeks old) were evaluated in 4 experimental procedures to study the addictive properties of sugar: (i) a drinking in the dark (DID) procedure to model sugar binging; (ii) a long-term free choice home cage drinking procedure measuring the sugar deprivation effect (SDE) following an abstinence phase; (iii) a long-term operant sugar self-administration with persistence, motivation and compulsivity measures and (iv) intracranial self-stimulation (ICSS). Female mice were more vulnerable to the addictive properties of sugar than male mice, showing higher binge and long-term, excessive drinking, a more pronounced relapse-like drinking following deprivation, and higher persistence and motivation for sugar. No sex differences were seen in a compulsivity test or reward sensitivity measured using ICSS following extended sugar consumption. This study demonstrates the occurrence of an addictive-like phenotype for sugar in male and female mice, similar to drugs of abuse, and suggests sex-dependent differences in the development of sugar addiction.

## Introduction

The terms substance use and addictive behaviors encompass disorders that can develop following the use of psychoactive substances. However, the term addiction is increasingly also applied to a range of other problematic behaviors, such as excessive gaming behavior. Consequently, the new psychiatric classification systems ICD-11 has now categorized drug addictions *vs.* behavioral addictions^1^. This marks a milestone in diagnosis and psychiatry. Despite the newly introduced behavioral addictions into ICD-11, there is public awareness that other behaviors such as excessive food/sugar consumption^2^ can also lead to addiction. However, the notion of a scientifically-defined “food/sugar addiction” remains a subject of debate. It has been suggested that non ICD-11 classified behavioral addictions should be classified using terms such as "problematic use" and described in their sociocultural context rather than within an addiction framework^2^.

It has been proposed that the problematic use of sugar, similar to chronic drug use, can lead to a complex behavioral disorder involving multiple interactions between genetic, biological and environmental aspects. In fact, the behavioral phenotypes associated with chronic drug abuse and problematic sugar consumption are similar with respect to compulsive overconsumption, craving, loss of control, and even withdrawal responses^2,3^. Thus, specific foods, such as those that are rich in sugar, may be capable of promoting addiction-like behavior and neuronal change under certain conditions, for example following a restriction/binge pattern of sugar consumption^4,5^. Importantly, the addictive properties of alcohol or drugs of abuse also develop after a restriction/binge pattern of consumption^6,7^. Thus, a critical factor in the development of alcohol or drug addiction is restriction/deprivation and multiple phases of deprivation and binging that can lead, at least in some individuals, to addictive behavior^6,7^.

There have been several attempts to model different aspects of excessive sugar consumption in rodents. For example, the repeated, excessive intake of sugar has been shown to create a state in which the administration of an opioid antagonist resulted in behavioral and neurochemical signs of withdrawal^8^. Furthermore, rats that consumed sucrose were more active in response to psychostimulants than control animals, as sugar intake caused cross-sensitization to amphetamine^9^, and locomotor sensitization to cocaine developed more rapidly in female mice drinking sucrose than in water consuming controls^10^. The repeated, excessive intake of sugar can also lead—following deprivation—to a sugar deprivation effect (SDE)^11^, which is considered a relapse-like phenomenon in the addiction field^7,12^. Several more aspects of repeated, excessive intake of sugar on behavioral and cognitive function^13^ have been studied in rodents; however, a comparative study on sex differences in different paradigms and animal models used in the addiction field has so far not been conducted.

Epidemiological studies have observed significant gender-specific differences in patients with addiction disorders, with females more vulnerable to the initiation of drug use and the development of excessive chronic and even compulsive behavior^14,15^. Similarly, women are more prone to the excessive intake of sugar and a greater incidence of obesity^16,17^. Similar to humans, extensive preclinical research with laboratory animals has found sex differences across different drug-related addictive phenotypes and mechanisms, revealing that females are more vulnerable to the initiation of drug use and subsequent withdrawal and relapse stages^15,18^. While fewer studies have evaluated sex differences in food/sugar-related phenotypes, one hypothesis is that sex differences related to food addiction may occur in rodents and parallel those in humans^15,19^.

Here we systematically tested the addictive properties of sugar in male and female mice using established paradigms and models from the drug addiction field. (i) We applied the drinking in the dark (DID) procedure to model sugar binging. The DID procedure was originally developed to induce alcohol intoxication and model alcohol binging in mice^20,21^. (ii) We also used a long-term free choice home cage drinking procedure and studied the sugar deprivation effect (SDE) following an abstinence phase. The deprivation effect is a measure of consumption during a relapse-like situation in the addiction field^7,12^. (iii) We also introduced a long-term operant sugar self-administration paradigm and quantified three criteria for addictive behavior: persistence, motivation and compulsivity. These three criteria have been used to operationalize cocaine addiction in rats^22^ and are now increasingly applied to other addictions in preclinical research^7,23^. Finally, we tested brain reward function in mice chronically exposed to sugar using intracranial self-stimulation (ICSS).

## Results

### Experiment 1. Characterization of binge sugar drinking in male and female mice using the DID paradigm

**Figure 1:**
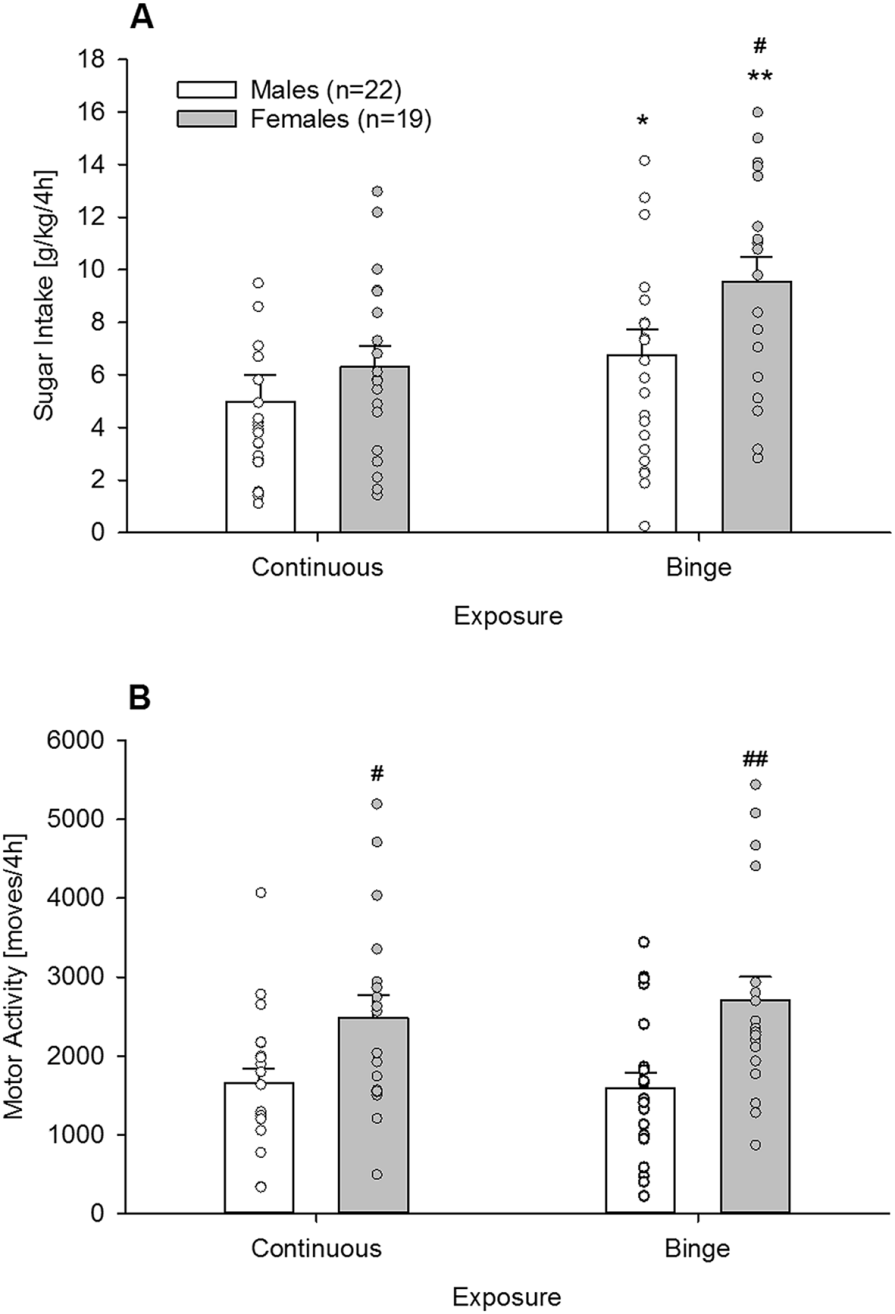
Characterization of sugar binge drinking and locomotor activity in male and female mice (Experiment 1). (**A**) During binge exposure (restricted sugar consumption during the first 4 h into the night active period) sugar intake was significantly higher in male (white bars, n = 22) and female (grey bars, n = 19) mice compared to a continuous free choice access. (**B**) Locomotor activity did not differ between binge and continuous sugar exposure. However, females displayed a general increased locomotion compared to males. All data represent mean + SEM. (*), (**) indicate *P* < 0.01 and 0.001versus continuous exposure, respectively; (#), (##) indicate *P* < 0.05 and 0.01 versus male mice, respectively.

Here we defined sugar binge drinking as the intake of an excessive amount of sugar in a short period. Thus, the amount of restricted sugar intake (in g/kg) consumed during the first 4 h of the dark period on the fourth day significantly exceeded unrestricted free choice sugar consumption in the same animals (Fig. 1A, Two-way ANOVA, *Exposure* effect: F_(1,39)_ = 31.4; *P* < 0.0001; *Sex* effect: F_(1,39)_ = 2.7; *P* = 0.1). Although there was no exposure x sex interaction (F_(1,39)_ = 2.7; *P* = 0.1) the main effect of exposure was further analyzed by performing one-way ANOVA by sex for continuous access and binge exposure separately. Although continuous free choice access did not differ between sexes (F_(1,39)_ = 1; *P* = 0.3), females showed a significantly higher intake during binge exposure compared to males (F_(1,39)_ = 4.3; *P* < 0.05; male mice: 5,0 g/kg, 95% CI 3.1–6.9 vs. female mice: 9.6 g/kg, 95% CI 7.8–11.3; |*d*|= 1.04).

When analyzing locomotor activity, we found that binge sugar access did not have any effect (Fig. 1B, *Exposure* effect: F_(1,39)_ = 0.2; *P* = 0.7). However, females displayed a general increased locomotion compared to males (Fig. 1B, *Sex* effect: F_(1,39)_ = 11.9; *P* < 0.05; post-hoc *P* < 0.05 and 0.01 during continuous and binge, respectively).

### Experiment 2a. Characterization of acquisition and maintenance of sugar drinking in male and female mice

**Figure 2:**
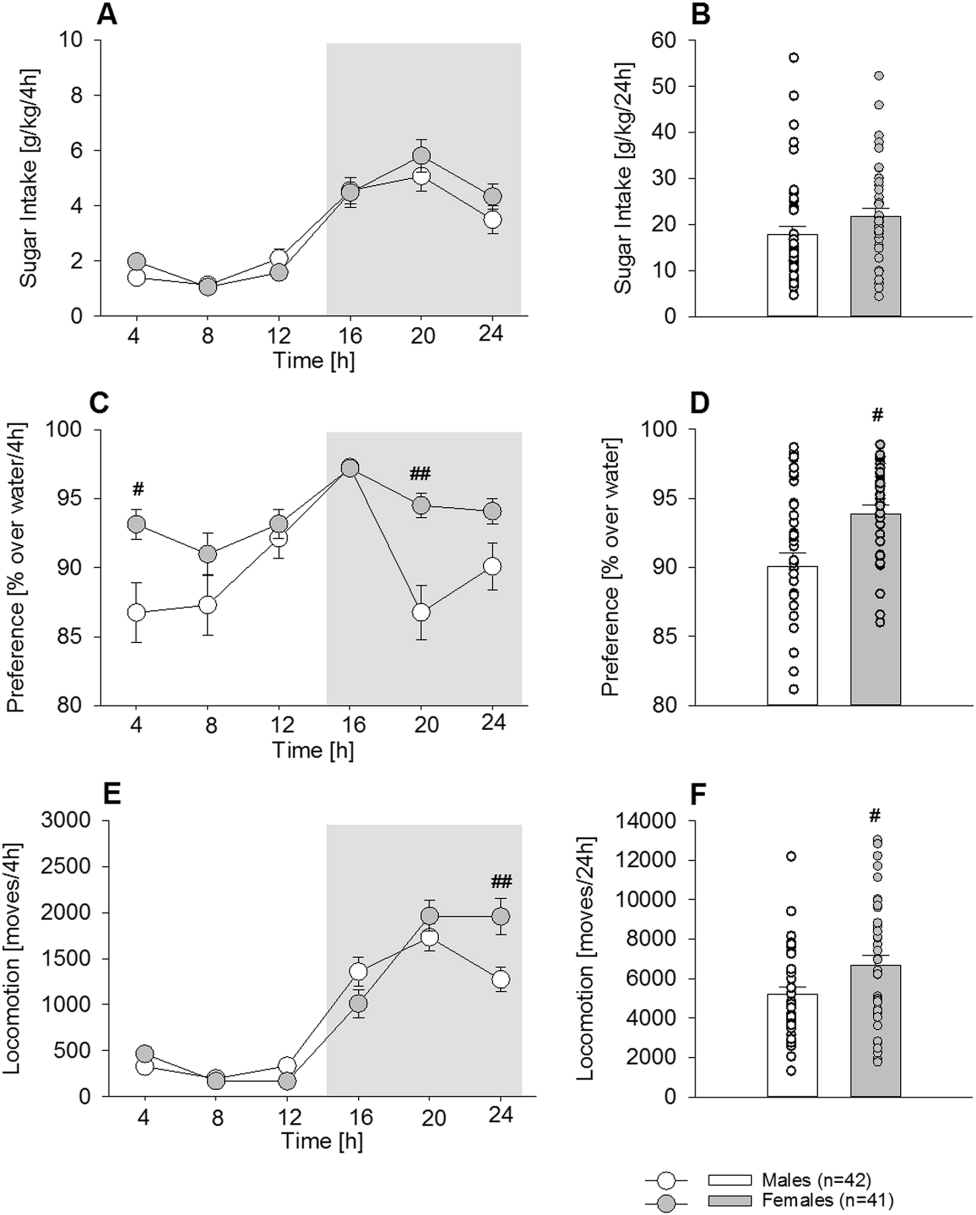
Acquisition of sugar consumption in male (n = 42) and female (n = 41) mice (Experiment 2a). Sugar drinking (**A**,**B**), preference over water (**C**,**D**) and locomotor activity (**E**,**F**) during the first 3 days of free choice sugar exposure. (**A**) Both males (white circles/bars) and females (grey circles/bars) showed the typical diurnal rhythmicity, with higher drinking levels during the dark, active phase compared with the light, inactive phase of the day with no differences between males and females (all day points differed significantly from all night points, not indicated). (**B**) Consequently, the total sugar intake during 24 h was similar in both groups. (**C**) Such rhythmicity was not that marked in the daily preference over water but the mean showed a clear diurnal pattern, with females showing higher preference between the end of the active and beginning of the inactive phase, (**D**) which resulted in a higher total preference compared to males. (**E**) Similar to the intake pattern, locomotor activity also showed the typical diurnal rhythmicity (all day points differed significantly from all night points, not indicated). Females showed higher locomotor activity which was restricted to the active phase. (**F**) As a result, the total daily locomotor activity was higher in females than in males All data represent means ± SEM. (#), (##) indicate *P* < 0.05 and 0.001 versus male mice, respectively.

During the acquisition phase (the first 3 days of free-choice sugar drinking), both males and females showed diurnal rhythmicity, with higher drinking levels during the dark, active phase compared to the light, inactive phase of the day (Fig. 2A, two-way ANOVA, *Phase* effect: F_(5,405)_ = 77; *P* < 0.001), with no differences between males and females (*Sex* effect: F_(1,81)_ = 0.3; *P* = 0.5). Consequently, the total sugar intake during 24 h was similar in both groups (Fig. 2B, One-way ANOVA, F_(1,81)_ = 2.6; *P* = 0.1). Diurnal rhythmicity was also present in the daily preference over water (Fig. 2C). The mean of the first 3 days indicated a diurnal pattern (Fig. 2C, *Phase* effect: F_(5,405)_ = 10.4; *P* < 0.001), with females showing a higher preference than males during the first four hours of the active phase and 8 h following the inactive phase (*Sex* effect: F_(1,81)_ = 10.6; *P* < 0.01; *Sex x Phase* interaction effect: F_(5,405)_ = 2.8; *P* = 0.05; post-hoc *P* < 0.05 and 0.001 during active and inactive phases, respectively), which resulted in a higher total preference (94%) compared to males (90%) (Fig. 2D, F_(1,81)_ = 10.6; *P* < 0.005). Similar to the intake pattern, locomotor activity (Fig. 2E) also showed a typical diurnal rhythmicity (Fig. 2E, *Phase* effect: F_(5,405)_ = 40.5; *P* < 0.001), and females showed higher locomotor activity (*Sex* effect: F_(1,81)_ = 9.6; *P* < 0.01) that was restricted to the last four hours of the active phase (*Phase x Sex* interaction effect: F_(5,405)_ = 5.2; *P* = 0.001; post-hoc *P* < 0.001). As a result, total daily locomotor activity was increased in females compared to males (Fig. 2F, F_(1,81)_ = 5.9; *P* < 0.05).

**Figure 3:**
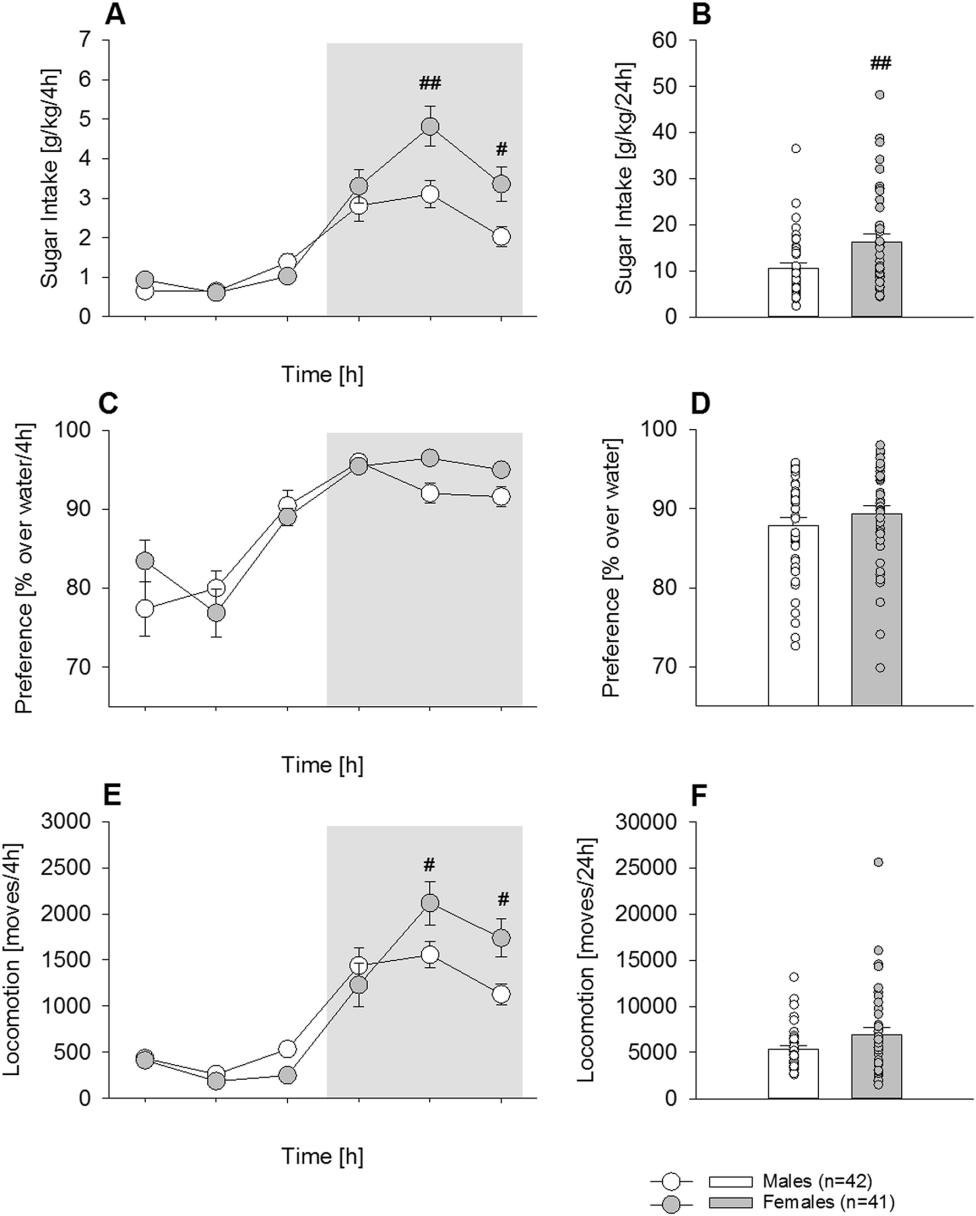
Chronic long-term sugar drinking in male (n = 42) and female (n = 41) mice (Experiment 2a). Sugar drinking (**A**,**B**), preference over water (**C**,**D**) and locomotor activity (**E**,**F**) during the last 3 days of a 6 weeks period of free choice sugar exposure in males (white circles/bars) and females (grey circles /bars). (**A**) There was a clear diurnal rhythmicity of sugar intake, but the females showed higher intake during the active phase. (**B**) As shown for 24 h intake, females consumed significantly more sugar than males. (**C**) The daily preference after long-term drinking showed a diurnal pattern and the mean taken of the last 3 days did not differ between both groups; total 24 h preference was also not different (**D**). (**E**) The locomotor activity remained high in the females and, similar to the acquisition phase, was restricted to the active phase. (**F**) However, the total daily locomotor activity did not differ between females and males. All data represent means ± SEM. (#), (##) indicate *P* < 0.05 and 0.01 versus male mice, respectively.

After a long-term drinking period of 8 weeks, the diurnal rhythmicity of sugar intake remained typical (Fig. 3A; *Phase* effect: F_(5,405)_ = 71.7; *P* < 0.001), but female mice showed higher intake during the last 8 h of the active phase (Fig. 3A, *Phase x Sex* interaction: F_(5,405)_ = 6.1; *P* < 0.001; post-hoc *P* < 0.05 and 0.01). In total 24 h intake, females consumed significantly more (53%) than males (Fig. 3B, F_(1,81)_ = 8.1; *P* < 0.01; male mice: 10.6 g/kg, 95% CI 8.6–12.6 vs. female mice: 16.2 g/kg, 95% CI 13–19.5; |*d*|= 0.65). In contrast to the pattern displayed during the acquisition phase, the daily preference following long-term drinking clearly had a diurnal pattern (Fig. 3C; *Phase* effect: F_(5,405)_ = 36.6; *P* < 0.001) that did not differ between both groups (*Sex* effect: F_(1,81)_ = 1.1; *P* = 0.3). Total 24 h preference was also not different (Fig. 3D, F_(1,81)_ = 1.1; *P* = 0.3). Locomotor activity (Fig. 3E) remained high in the females and, similar to the acquisition phase, was restricted to the last 8 h of the active phase (Fig. 3E, *Phase*: F_(5,405)_ = 64; *P* < 0.0001, *Gender*: F_(1,81)_ = 0.6; *P* = 0.4; post-hoc *P* < 0.05).This increase was significant for total 24 h activity (Fig. 3F, F_(1,81)_ = 3.9; *P* = 0.05).

**Figure 4:**
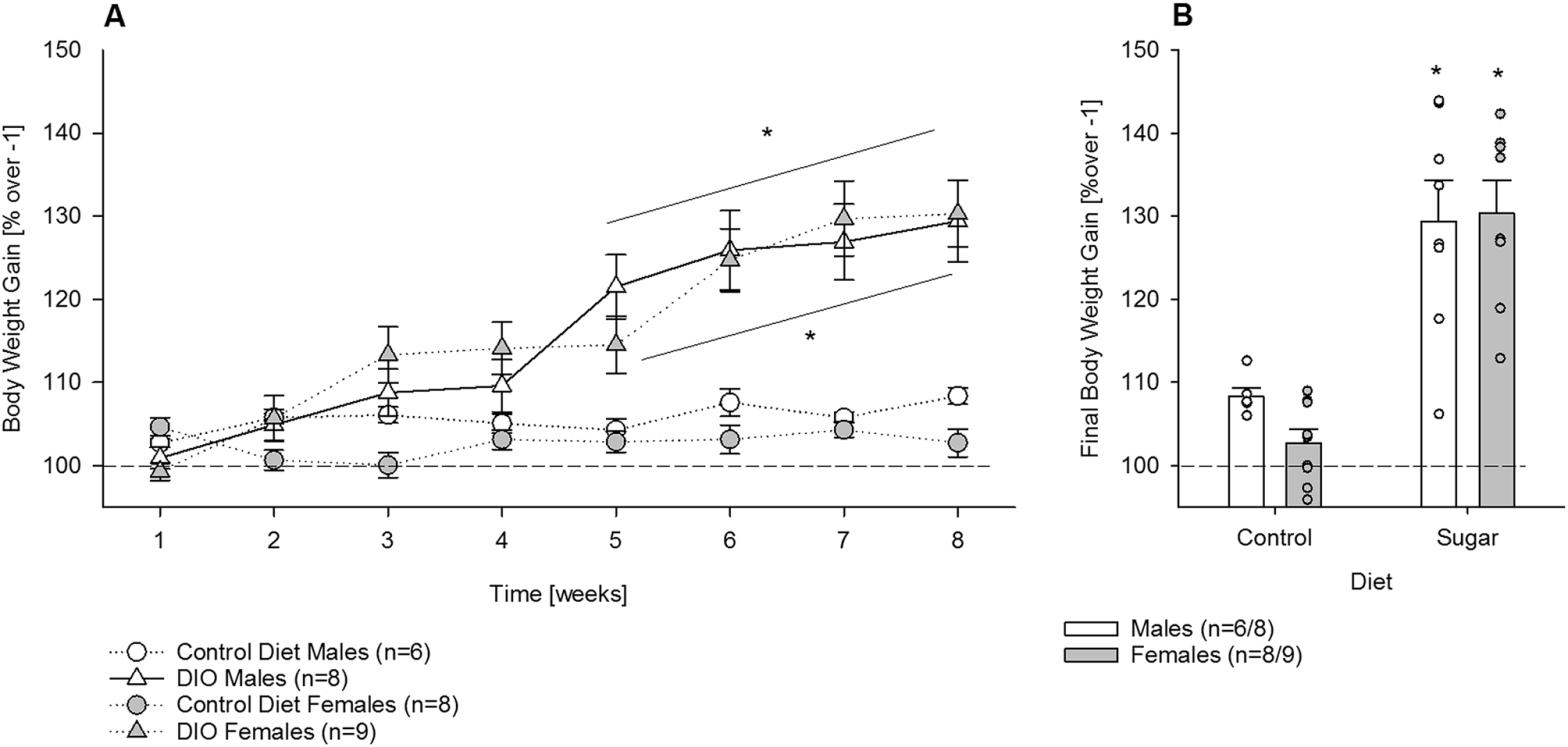
Pronounced increase in bodyweight during chronic long-term sugar drinking in male (n = 6/8) and female (n = 8/9) mice (Experiment 2). (**A**) Compared to the sugar-free week before (week -1), chronic sugar intake over 8 weeks (males, white triangle; females, grey triangle) led to a significantly higher increase in bodyweight than in the water drinking control group (males, white circle; females, grey circle) but no sex differences were observed. (**B**) After 8 weeks, the total body weight increase was similar in males (white bar) and females (grey bar) and significantly increased compared to the control group. All data represent means ± SEM. (*) indicate *P* < 0.001 versus water control group.

Importantly, chronic sugar intake over 8 weeks led to a significant increase in body weight when compared to the sugar-free period one week prior (Fig. 4A, Two-way ANOVA; Fig. 4A,B *Sugar* effect*:* F(1,27) = 31.1; *P* < 0.001; *Time* effect*:* F(7,189) = 61.5; *P* < 0.001), which was significant from the 5th week on (post-hoc *P* < 0.001), but no sex differences were observed (*Sex* effect: F(1,27) = 0.35; *P* = 0.56). After 8 weeks, the total body weight increase was similar in males and females (Fig. 4B, *Sex* effect*:* F(1,27) = 0.5; *P* = 0.5) and significantly increased compared to the control group (*Sugar* effect*:* F(1,27) = 55.7; *P* < 0.001).

### Experiment 2b. Characterization of relapse-like sugar intake (sugar deprivation effect (SDE) in male and female mice

**Figure 5:**
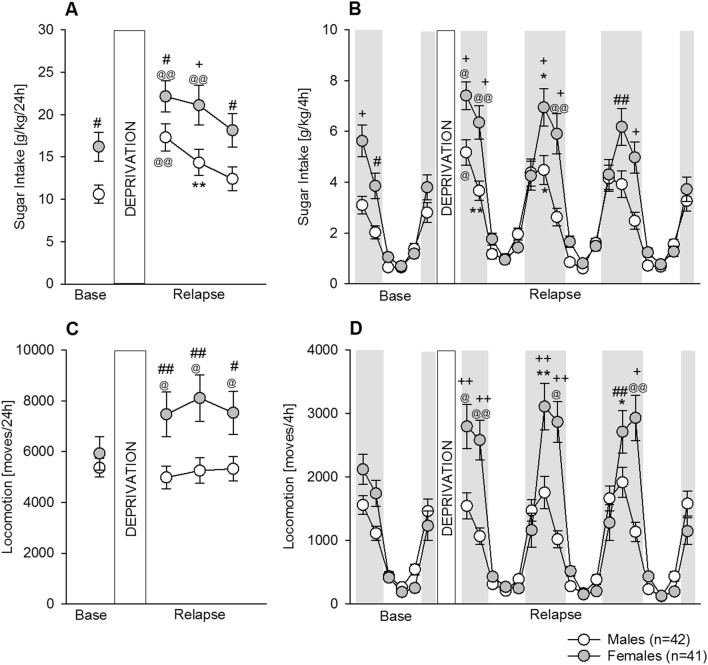
Characterization of the sugar deprivation effect (SDE) in male and female mice (Experiment 2b). (**A**) A period of sugar deprivation significantly increased the sugar intake in both male (white circles, n = 42) and female (grey circles, n = 41) mice*,* indicative of a sugar deprivation effect (SDE), which lasted 2 days, and was higher in females. (**B**) During the first day, dissection of the SDE into 4 h intervals showed that intake was strongly pronounced during the first 4 h of sugar re-exposure and lasted for 8 h in both males and females. During the second day, the SDE was stronger in females compared to males, as it lasted for 8 h, while in males it was attenuated in respect to the first day and lasted only 4 h. (**C**) Females (but not males) showed a very pronounced increase in locomotor activity which lasted 3 days. (**D**) Dissection of the locomotor activity into 4 h intervals showed that it was strongly pronounced during the first 4 h of sugar re-exposure and lasted not longer than 8 h in females. All data represent means ± SEM. (#, ##, + , + +) indicate *P* < 0.05; 0.01; 0.005; 0.001 vs male mice; respectively; (*, **, @,@@) indicate *P* < 0.05; 0.01; 0.005; 0.001 vs corresponding baseline point, respectively.

After 8 weeks of home cage voluntary sugar consumption, all animals underwent a 15-day period of sugar deprivation that significantly increased the 24 h sugar intake relative to the baseline (mean of the last 3 days) in both male and female mice*,* indicative of a sugar deprivation effect (SDE, Fig. 5) that lasted 2 days (Fig. 5A, two-way ANOVA; *Deprivation* effect: F_(3,243)_ = 23.9; *P* < 0.0001; post-hoc *P* < 0.001 during the first day and *P* < 0.01 and 0.001 during the second day for male and females, respectively) and was higher in females (*Sex* effect: F_(1,81)_ = 6.9; *P* < 0.05; post-hoc *P* < 0.05 and 0.005 during the first and second day; male mice: day 1—17.3 g/kg, 95% CI 14.3–20.4, day 2—14.3 g/kg, 95% CI 11.4–17.3 vs. female mice: day 1—22.3 g/kg, 95% CI 18.6–25.7, day 2—21.1 g/kg, 95% CI 16.6–25.6; male vs female mice for both days |*d*|= 0.45). During the first day, the division of the SDE into 4 h intervals showed that the SDE was strongly pronounced during the first 4 h of sugar re-exposure and lasted not longer than 8 h in both males and females (Fig. 5B, *Deprivation* effect: F_(23,1863)_ = 74; *P* < 0.0001). Post-hoc analysis indicated *P* < 0.05 and 0.01 in males and *P* < 0.005 and 0.001 in females. During the first 8 h, females also differed significantly from males (post-hoc P < 0.005). Indeed, during the second day, the SDE was stronger in females than males (*Sex* effect: F_(1,81)_ = 6.8; *P* < 0.05; post-hoc *P* < 0.005 vs. males during the first 8 h), again lasting 8 h (post-hoc *P* < 0.05 and 0.001 vs. baseline), while in males it was attenuated with respect to the first day and lasted only 4 h (*Sex x Deprivation* effect: F_(3,243)_ = 7.7; *P* < 0.0001).

Locomotor activity analysis also showed a very pronounced increase in females that lasted 3 days (Fig. 5C). This increase was not only significant compared to baseline (*Deprivation* effect: F_(3,243)_ = 4.1; *P* < 0.01; post-hoc *P* < 0.05), but also strongly differed across sex (*Sex* effect: F_(1,81)_ = 5.9; *P* < 0.05; *Sex x Deprivation* interaction effect: F_(3,243)_ = 5.4; *P* < 0.01; post-hoc *P* < 0.05 and 0.01). Almost identical to the intake pattern, separation of the locomotor activity into 4 h intervals showed that it was strongly pronounced during the first 4 h of sugar re-exposure (Fig. 5D, *Deprivation* effect: F_(23,1863)_ = 63.3; *P* < 0.0001) and lasted not longer than 8 h in females (Fig. 5D, *Sex*: F_(1,81)_ = 5.8; *P* < 0.05 and *Sex x Deprivation*: F_(3,243)_ = 12.2; *P* < 0.0001). Post-hoc analysis indicated significant differences in the first 8 h during all 3 days compared to baseline and relative to males.

### Experiment 3a. Characterization of long-term operant self-administration of sugar in male and female mice

**Figure 6:**
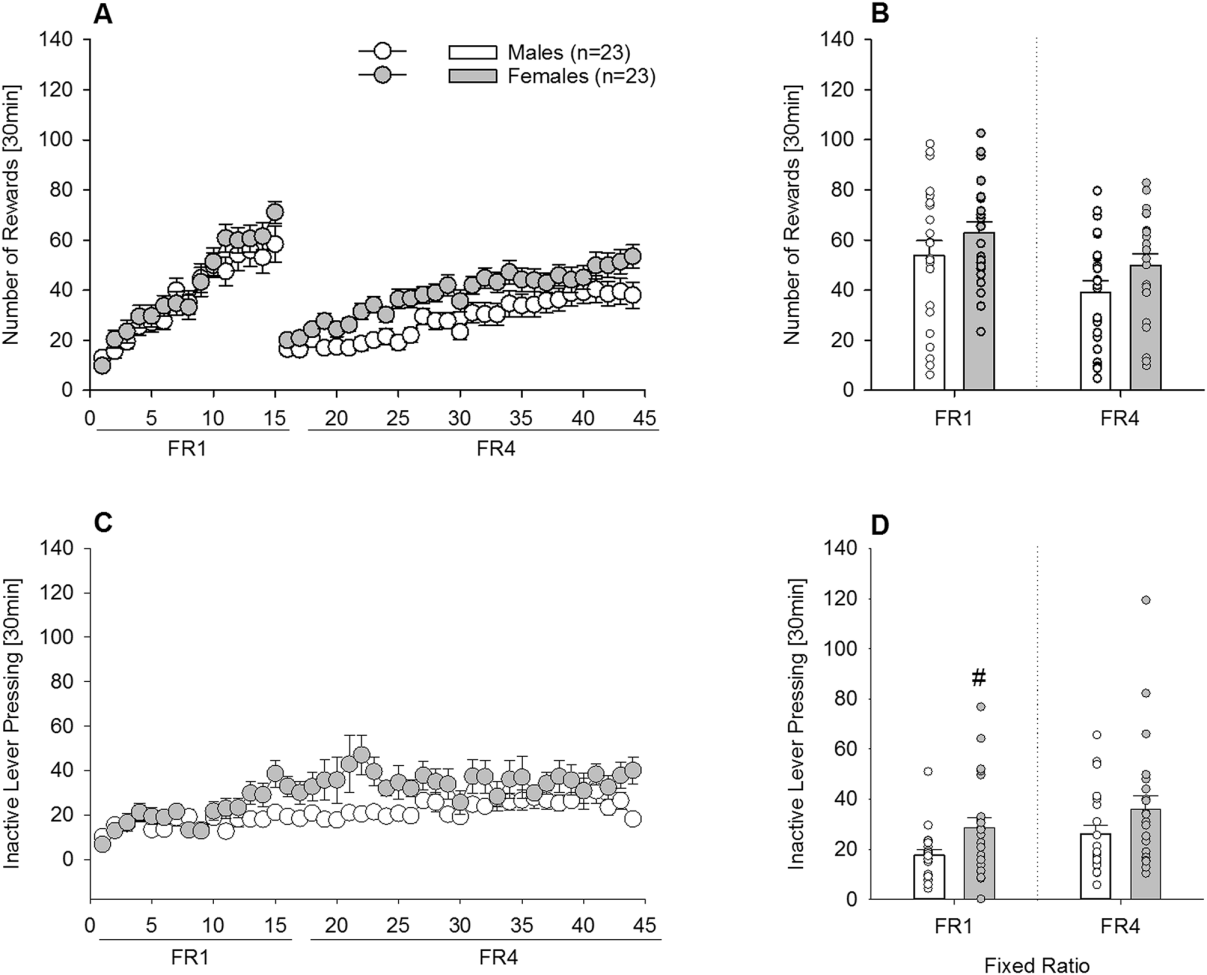
Long-term operant self-administration for sugar in male (n = 23) and female (n = 23) mice (Experiment 3a). (**A**) Number of rewards earned, (**C**) inactive lever pressing per sessions, during FR1 (15 sessions) and FR4 (29 sessions) schedule of reinforcement. Mean from the last 5 sessions of (**B**) rewards earned, and (**D**) inactive lever pressing in males (white circles/bars) and females (grey circles/bars). (**A**) During the FR1 phase, all mice similarly acquired and maintained stable active lever pressing for sugar and (**B**) the mean from the last 5 sessions did not differ between male and female mice. (**C**) Inactive lever pressing was higher in female mice across the 15 FR1 sessions, and (**D**) the mean of the last 5 sessions showed a significant increase compared to male mice. During the FR4 phase, (**A**) females earned more rewards and made more responses on the inactive lever (**C**,**D**). All data represent mean ± SEM. # indicates *P* < 0.05 versus male mice.

A third group of mice was trained to self-administer sugar. During the FR1 phase, all mice similarly acquired and maintained stable active lever pressing for sugar (Fig. 6A), and the mean of the last 5 sessions did not differ between male and female mice (Fig. 6B, F_(1,44)_ = 1.6; *P* = 0.2). However, as observed in Fig. 6C, inactive lever pressing was higher in female mice across the 15 FR1 sessions, and the mean of the last 5 sessions showed significant differences compared to male mice (Fig. 6D; F_(1,44)_ = 6.2; *P* < 0.05). During the FR4 phase, the overall performance of the females was higher than males: females earned more rewards and made more responses on the inactive lever (Fig. 6A,C); however, the mean of the last 5 days did not result in a significant difference between sexes (Fig. 6B: F_(1,44)_ = 2.8; *P* = 0.1; and Fig. 6D: F_(1,44)_ = 2.4; *P* = 0.1).

### Experiment 3b. Characterization of an addiction-like phenotype for sugar in male and female mice

**Figure 7:**
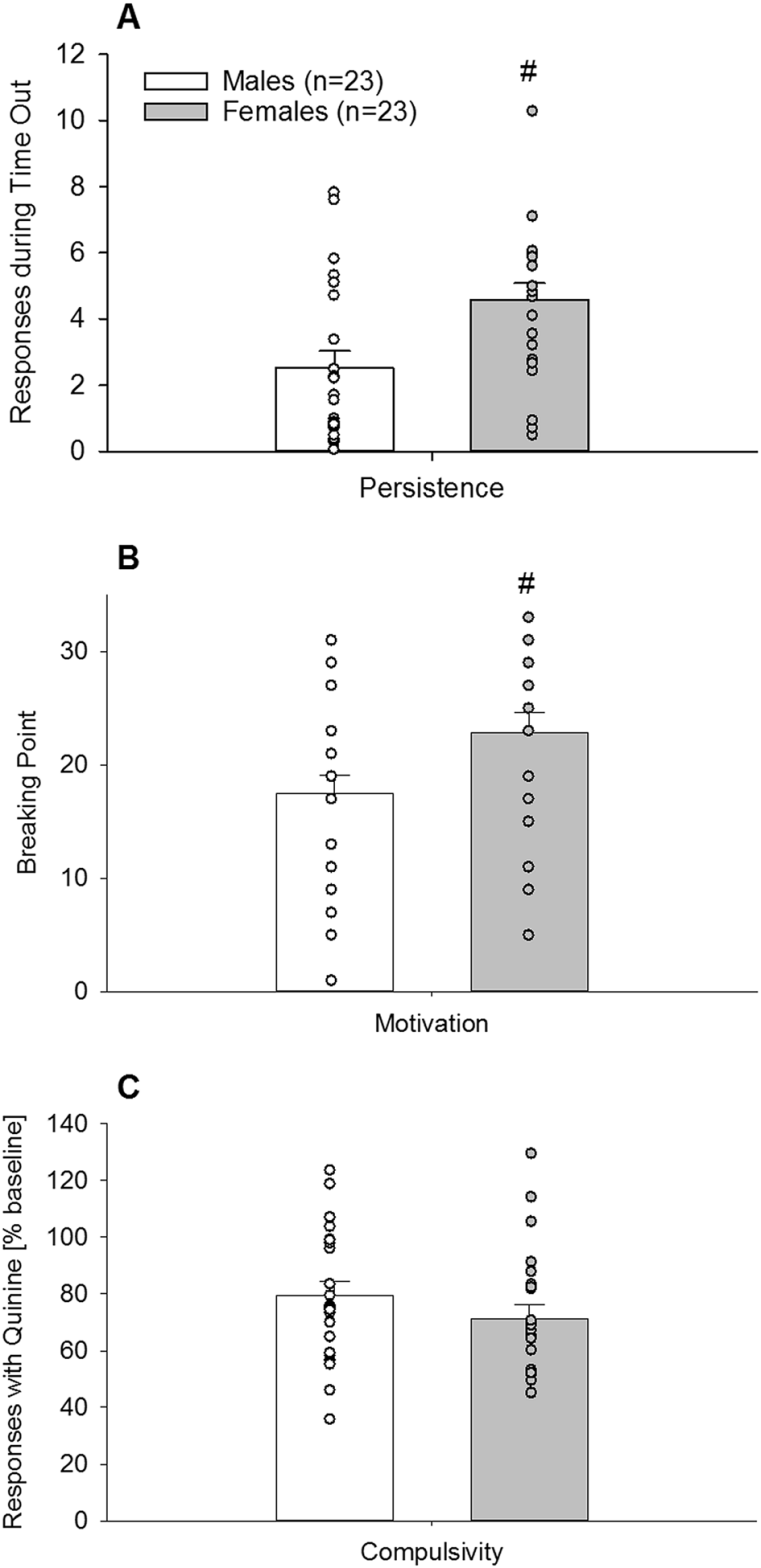
Addictive-like phenotypes of persistence, motivation and compulsivity in long-term operant sugar self-administering male (n = 23) and female (n = 23) mice (Experiment 3b). (**A**) Persistence was measure by the TO test. During the single 6 min time out of sugar access, the number of lever presses per min was almost doubled in females (grey bar) compared to male (white bar). (**B**) Motivation as assessed by a PR schedule of reinforcement. The breakpoint or limit to the amount lever pressing that male mice were willing to perform to obtain sugar was around 17, while in females it was significantly increased to almost 23. (**C**) Compulsive-like behavior by taste adulteration with quinine reduced the number of lever presses to obtain sugar similarly in both male and female mice. All data represent mean + SEM. (#) and (##) indicate *P* < 0.05 and *P* < 0.01 versus male mice, respectively.

Next, the addictive-like phenotypes of persistence, motivation, and compulsivity were studied using the time out (TO), progressive ratio (PR), and quinine tests, respectively (Fig. 7). During the TO test, the number of lever presses per min was almost doubled in females compared to male mice (Fig. 7A, F_(1,44)_ = 8.8; *P* < 0.005), indicating higher persistence for sugar-seeking (|*d*|= 0.89). Motivation was assessed using a PR schedule of reinforcement. As illustrated in Fig. 7B, females demonstrated a significantly higher breakpoint than males (F_(1,44)_ = 5; *P* < 0.05), indicating a higher motivation (|*d*|= 0.68). The final test measured compulsive-like behavior using taste adulteration with quinine (Fig. 7C). A 0.8 mM quinine concentration added to the sugar solution reduced the lever pressing to obtain sugar similarly in both male (79%) and female (71%) mice, and no significant differences were found (F_(1,44)_ = 1.5; *P* = 0.2). Thus, in contrast to persistence and motivation, compulsive-like behavior for sugar was not higher in females compared to male mice.

### Experiment 4. Characterization of reward sensitivity by ICSS in male and female mice

**Figure 8:**
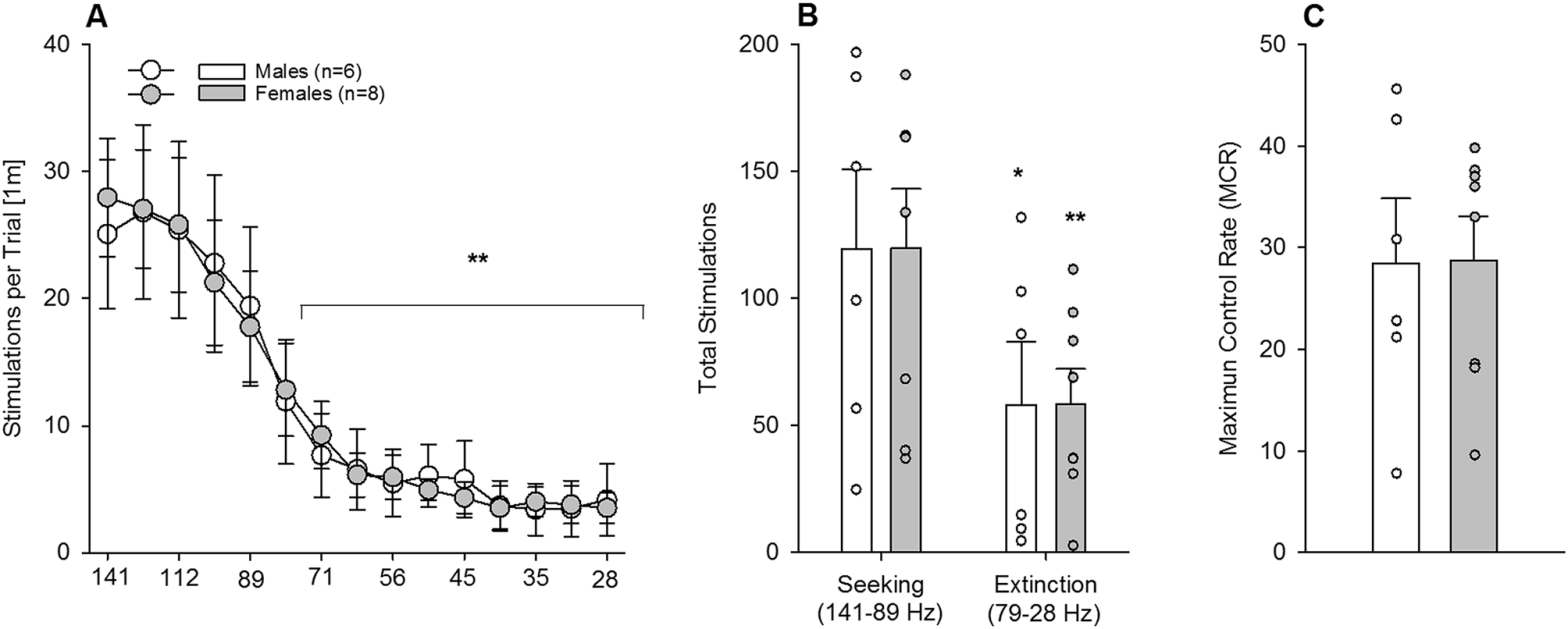
Characterization of brain reward sensitivity in male (n = 6) and female (n = 8) mice (Experiment 4). (**A**) Following ICSS training males (white circles) and females (grey circles) underwent the testing of 15 descending frequencies and all mice showed the expected frequency-dependent decrease in the number of stimulations per trial, which became significant from the 6th frequency on. (**B**) Both seeking (first 5 frequencies) and extinction (remaining 10 frequencies) components were almost identical in all mice, which were characterized by a decrease in the extinction phase, compared to the seeking phase. (**C**) Likewise, all mice also showed an indistinguishable reinforcement rate (males, white bars; females, grey bars). All data represent mean ± SEM. (*) and (**) indicate *P* < 0.05 and *P* < 0.01 versus first 5 frequencies (**A**) or seeking (**B**), respectively.

We further tested a fourth group of male and female mice brain stimulation reward using the ICSS paradigm^24,25^. All mice showed the expected frequency-dependent decrease in the number of stimulations per trial, which became significant from the 6th frequency on (Fig. 8A, two-way ANOVA, *frequency* effect: F_(14,168)_ = 26.8; *P* < 0.0001; post-hoc *P* < 0.05). During the “seeking” and “extinction” components, the performance of both male and female mice was almost identical (Fig. 8B, *Sex* effect (F_(1,12)_ = 0.00; *P* = 1) and was characterized by a significant 40% reduction during the “extinction” component compared to the “seeking” component (*Component* effect (F_(1,12)_ = 16; *P* < 0.01). Consistent with these findings, all mice showed an indistinguishable reinforcement rate (Fig. 8C, F_(1,12)_ = 0.00; *P* = 1).

## Discussion

We performed three experiments to examine and quantify addictive features of repeated, excessive sugar consumption and one experiment to measure the reward sensitivity in male and female sugar-experienced C57BL6/N mice. In the first experiment, we show using the DID procedure that female mice exhibited augmented binge-like intake of sugar relative to male mice. In the second experiment, we show that following long-term free choice sugar consumption, female mice had a higher intake than male mice, especially during the active night phase. A deprivation phase resulted in an SDE that was again more pronounced in female mice, and accompanied by a strong psychomotor stimulation. In the third experiment, we demonstrated enhanced persistency and motivation for sugar in female mice, but no sex difference in a compulsivity test. In experiment 4, we show that long-tern sugar consumption does not lead to sex-specific alteration in brain reward stimulation.

Using the DID procedure^20,21^, sugar binging over a restricted 4 h period was induced in both sexes, with a consumption of 50–75% greater than consumption during continuous 24 h access. Although there is no consensus on the criteria defining a binge episode in rodents (i.e., hyperphagia, loss of control or/and duration of the event), it has been suggested that a two-fold increase in caloric intake relative to controls appears to be a reasonable criterion to determine hyperphagia^26^; thus, mice in the DID procedure would fulfill the criterion of hyperphagia and show sugar binge consumption. Females showed a more significant increase than males relative to continuous drinking, as well as higher binge drinking. Locomotor activity under continuous sugar exposure was higher in females, and interestingly, this difference increased during binge conditions. This augmented activity can be seen as enhanced psychomotor stimulation, which is characteristic of a reward-seeking response^27^.

We also studied long-term free choice sugar consumption. To date, chronic sugar consumption has almost exclusively been used to induce obesity in mice. In the diet/(sugar)-induced obesity model, the consequences on metabolic diseases or particular organ damage (i.e., liver) are of particular interest. However, the consequences of long-term free choice sugar consumption are usually not discussed in the context of addiction. Here we followed our standard protocol of long-term ethanol drinking in mice^28^ and transferred it to voluntary sugar intake. As previously observed with alcohol, one key aspect involved in sugar drinking is control by the circadian clock, resulting in diurnal rhythmicity of drinking behavior. Thus, regardless of short or long-term exposure, mice voluntarily consumed more sugar during the active, dark phase, than in the inactive, light phase. This diurnal rhythmicity remained stable over the entire time course of the experiment, including a deprivation phase. In comparison, following long-term alcohol or drug exposure and deprivation, disturbances in diurnal rhythmicity have been observed^6,29^, which may be a distinction between sugar and drug addictive-like behaviors.

After 6 weeks of long-term free choice sugar consumption, female mice showed an approx. 50% higher sugar consumption than males. These findings are consistent with what has been reported with alcohol in mice^30^ and other drugs of abuse, in which female mice consume higher amounts of drugs under long access procedures^31^.

Following long-term free choice sugar consumption, a deprivation phase was introduced to induce relapse-like drinking, referred to here as the sugar deprivation effect (SDE)^11^. Sugar deprivation for 2 weeks induced a robust SDE in all mice that was most pronounced during the first 4 h after re-exposure to the sugar solution, similar to what we have previously found during the alcohol deprivation effect (ADE) in mice^28^. However, the SDE had a longer duration, as the increased intake persisted for at least two days, in contrast to the short-lasting ADE in mice (approx. 8 h). This suggests that in mice, sugar deprivation may induce a higher “craving state” than alcohol. Furthermore, our data also indicate that females had a higher craving for sugar than males, as shown by the enhanced SDE. Moreover, during the second day, during which the intake slightly decreased in males, females had an almost identical consumption compared to the first SDE day. This phenotype seems to be specific for sugar, as we have previously shown that females do not show an ADE after a period of deprivation, probably due to high basal intake. Thus, excessive baseline drinking usually results in decreased consumption, a phenomenon referred to as an inverse ADE^12,30^ that has been reported in various alcohol-preferring rat lines and high alcohol-preferring C57BL/6J mice [i.e.,^32,33^].

Another phenotype in female mice usually not observed with alcohol was a pronounced increase in locomotor activity during the SDE, which persisted up to 3 days. Thus, excessive sugar consumption may lead to enhanced psychomotor stimulation, a phenomenon seen after the intake of psychostimulants and in patients with attention deficit hyperactivity disorder (ADHD) caused by a disruption in dopamine signaling resulting from a reduction in dopamine D2 receptors in reward-related brain regions^34^. A similar mechanism may be in place in sugar binging, as rats show reduced mRNA levels for the D2 dopamine receptor in the brain reward system^35^.

We further applied operant sugar self-administration and quantified three criteria for addictive-like behavior—persistence, motivation and compulsivity. We found that female mice exhibited enhanced persistence and motivation—as measured by responding during the time out period and PR tests, respectively—compared to males. In contrast, there was no sex difference in compulsivity, as measured by quinine taste adulteration. These findings are consistent with rodent studies demonstrating that cue-reward learning in self-administration paradigms with drugs of abuse or natural rewards is more pronounced in females than in males^36–38^.

Finally, we used brain stimulation to determine whether the phenotype observed in females may be related to sugar-induced alterations in brain reward system sensitivity. Following long-term excessive sugar consumption, mice were tested for ICSS, which resulted in no difference between males and females. Thus, innate sex differences in the brain reward system do not contribute to the enhanced vulnerability for sugar in female mice, and long-term excessive sugar consumption in mice does not appear to alter reward processing. A potential limitation of the ICSS experiment was the lack of baseline testing of the mice prior to sugar exposure, which is a technically demanding experiment due to long-term electrode maintenance (8 weeks). Furthermore, since previous ICSS studies do not report any sex differences under naïve baseline conditions^39,40^, we found it reasonable not to test ICSS prior to sugar exposure.

In conclusion, our work suggests the occurrence of a sugar-related addictive-like phenotype in mice, similar but to some extent also different than that resulting from drugs of abuse (e.g., no alterations in diurnal rhythmicity following excessive sugar consumption and a longer duration of the sugar deprivation effect). Further studies are needed to determine whether these findings extend to different food components (i.e., sugary, salty and fatty foods) with respect to addictive-like behavior. Furthermore, we show enhanced vulnerability towards excessive sugar consumption, craving and relapse in female mice. Although more preclinical studies are needed, our finding of augmented excessive sugar consumption in female mice is consistent with a recent cross-sectional population-based study in more than 210,000 participants showing that women have higher intakes of sugar than men^16^. Whether women develop problematic sugar use more easily remains a matter of debate, but our study reinforces the idea that the occurrence of higher obesity rates in females may be due to the consumption of high sugar containing foods^41,42^.

## Methods

### Animals

For all four experiments, we used 8–10 week-old C57BL/6N mice. Mice were single-housed in standard hanging cages at 21 ± 1 °C and 50 ± 5% relative humidity on a reversed 12 h light/dark cycle, with lights on at 7:30 p.m. The animals were provided with standard rodent food (Altromin Spezialfutter GmbH & Co, LASQCdiet Rod16-H. Composition: cereals, vegetable by-products, minerals, oils and fats, yeast; crude nutrients: 16.30% crude protein, 4.30% crude fat, 4.30% crude fibre, 7.00% crude ash), a bottle containing 5% (w/v) sugar solution during the DID and long-term sugar paradigms (see below for details) and tap water ad libitum. We used a large sample size because the results presented here a part of a bigger study, including molecular and cellular studies. For that reason, we needed much more animals than were necessary for the behavioral experiments.

Before starting with the DID and long-term sugar exposure procedures, we first assessed the possible role of the caloric value or of taste sensitivity between males and females in a preliminary experiment. Mice were exposed for 4 h to different sugar concentrations (1, 3, 5, 10% solutions), and the intake was measured every 40 min. Females showed a higher intake of sugar at the lowest (1%) concentration tested. However, such a difference was not present anymore at a concentration of 3% or higher concentrated solutions (data not shown). Since 5% is the standard concentration used in operant procedures and no sex differences were seen at this concentration, we decided to use this concentration for all experiments.

All the experiments were performed in the dark cycle. All mice were handled on a daily basis before starting the experiments and were habituated to the behavioral testing environments. The study was carried out in compliance with the ARRIVE guidelines. Procedures for this study complied with the regulations covering animal experimentation within the European Union (European Communities Council Directive 86/609/EEC) and Germany (Deutsches Tierschutzgesetz) and the experiment was approved by the German animal welfare authorities (Regierungspräsidium Karlsruhe).

### Experiment 1. Binge sugar drinking: Drinking in the Dark (DID) paradigm

For the DID paradigm, 22 male and 19 female mice were used. We used a modified version of the standard DID protocol originally developed by Rhodes et al.^20^ to model binge consumption of sugar. In brief, during the first 3 days, 4 h into the dark cycle, animals had access for 2 h to standard food and a single bottle containing 5% (w/v) sugar solution and on the fourth day, mice were exposed for 4 h under identical conditions (food and sugar). Sugar solution was freshly prepared daily, and sugar consumption was calculated using the Drinkometer system (see below). Data collected during the 4-h DID period were expressed as g/kg body weight for sugar intake. Following the DID, all animals had 24 h access to sugar during the next 3 days and the amount consumed during both conditions was used for statistical analysis.

### Experiment 2. Home cage two-bottle free choice sugar drinking and assessment of relapse-like drinking by means of the sugar deprivation effect (SDE)

For this experiment, 42 male and 41 female mice were used. Mice had continuous free choice access to a bottle containing a sugar solution (5% w/v) solution and a bottle with tap water in the homecage for 8 weeks. During the first and last 3 days of sugar exposure, sugar and water intake was recorded using the Drinkometer system^30,43^. Mice were afterwards deprived from sugar for 12–15 days, during which they only had access to two bottles of tap water. After the deprivation period, the SDE was tested for 72 h by reintroducing the sugar bottle.

Sugar (g/kg) and water (ml) intake as well as the sugar preference (% of total fluid intake) were calculated per day. During baseline and SDE measurements, sugar and water intake was additionally calculated in 4 h time intervals. Baseline sugar and water intake was calculated as the mean of the last 3 d of baseline recording.

### Experiment 3. Long-term operant self-administration and assessment of motivation, persistence and compulsivity for sugar consumption

Twenty-three male and 23 female C57BL/6N mice were used as for the operant sugar self-administration. Mice were trained and tested in operant chambers (TSE Systems, Bad Homburg, Germany). Each chamber had two ultrasensitive levers (required force, ≤ 1 g) on opposite sides: one functioning as the active and one as the inactive lever. Next to each lever, a front panel containing the visual stimulus was installed above a drinking microreservoir. When the programmed ratio requirements were met on the active lever, 10 μl of the 10% sugar solution was delivered into a microreservoir, and the visual stimulus was presented via a light located on the front panel. Responses on the inactive lever were recorded but had no programmed consequences.

Mice were trained to self-administer 10% sugar (w/v) in 30 min daily sessions on a fixed ratio (FR) 1 schedule of reinforcement, where each response resulted in delivery of 10 µl of fluid. Following a lever press a 5 s time-out period was in effect, during which responses were recorded but not reinforced. After 15 sessions, the response requirements were enhanced to a FR4 schedule. After 30 sessions under FR4 responding and the establishment of stable baseline responding mice were tested for an addictive-like phenotype.

At the end of the training, we measured first the persistence, then the motivation, and finally the compulsivity for sugar self-administration. The persistence of response was measured using the time out (TO) test as described previously^44,45^. This 30-min session was composed of two 12-min available periods separated by one 6-min unavailable period as “time-out’’, during which the house light went on and active pressings resulted in no consequences except being registered. The tests were repeated for another three times, of which the first session was considered as the habituation to novelty, and the mean value of the last three sessions were calculated as a measure for persistence in responding for sugar when sugar was not available. Motivation for sugar was measured by a progressive ratio (PR) schedule of reinforcement in which the response requirement (the number of lever responses required to receive the reward) was progressively increased: the reinforcement schedule increased consecutively from ratio 1 by step size 2 for each reward. The last accomplished ratio was taken as the breaking point. Sessions were ended when mice were not earning a reward for 30 min. Compulsive-like behavior or resistance to aversive stimuli was measured by taste adulteration with quinine. A 0.8 mM quinine concentration was added to the sugar solution and the percentage of reduction over baseline was taken as a measure of compulsivity. Between the different test situations, mice were measured for 3–4 days for their baseline self-administration.

### Experiment 4. Assessment of brain stimulation reward by intracranial self-stimulation (ICSS)

The last experiment involved 6 male and 8 female sugar experienced mice. In order to assess the function of the brain reward system, ICSS was performed as described previously^24,25^ in a subset of mice from experiment 2 (continuous free choice access to a bottle containing a sugar solution (5% w/v) solution and a bottle with tap water in the home cage for 8 weeks). Briefly, mice were anesthetized with 1.5–1.8% of isoflurane (CP-Pharma, Burgdorf, Germany), stereotaxically implanted with insulated monopolar stainless steel electrodes (0.28 mm diameter) (Plastics One, USA) to the right medial forebrain bundle in the lateral hypothalamus (coordinates from Bregma: anterior (AP) − 1.2, lateral (ML) + 1, ventral (DV) − 5.4), and trained to respond for brain stimulation reward (BSR). Three days after surgery mice were trained to respond for brain stimulation reward (BSR). Mice spun a wheel fixed on the wall to receive contingent stimulations using an intracranial self-stimulation system (containing an operant box, a PHM-152 stimulator and the modules from Med Associates (Vermont, USA). Each ICSS box was enclosed in a ventilated sound-attenuating cubicle and connected to a controlling computer. During the training sessions, each response of one-quarter wheel turning resulted in the delivery of a 0.5-s square-wave cathodal pulse (100 µs per pulse). A 0.5-s timeout period simultaneous to a contingent stimulus light followed each reward, during which responding was recorded but had no consequences. The stimulation frequency was first fixed at 141 Hz, and the current intensity ascended by 10 µA every 10 min until the minimal amplitude that maintains a high and reliable reinforcement rate (≥ 30 rewards/min). Once the current intensity was determined, mice were trained with the rate-frequency testing sessions. During each testing session, mice responded during three consecutive series of 15 descending frequencies (0.05 log10 steps).

Each trial lasted for 60 s and began with 5 non-contingent primers (0.5 s long) followed by the 0.5-s timeout durations, after which a 50-s voluntary self-stimulation period occurred, which ended with a 5-s unavailability episode. Each effective response in the 50 s available period brought a hedonic stimulation together with a 0.5-s cue light presentation.

Maximum control rate (MCR), and total number of stimulations, were calculated from the mean of the second and third series. Stimulation seeking and extinction components were calculated from the total number of stimulations during the first 5 highest frequencies and the remaining 10, respectively.

### Statistics

Statistical analyses were performed by ANOVA with Newman–Keuls test for *post-hoc* comparisons using Statistica 10 (StatSoft). All values are given as mean ± SEM, and statistical significance was set at *p* < 0.05. For the main effects on sex differences, we also report mean values with 95% confidence intervals and effects sizes with Cohen’s d.
